# Characterization of the bacterial microbiota across the different intestinal segments of the Qinghai semi-fine wool sheep on the Qinghai-Tibetan Plateau

**DOI:** 10.5713/ab.20.0809

**Published:** 2021-06-23

**Authors:** Xungang Wang, Linyong Hu, Hongjin Liu, Tianwei Xu, Na Zhao, Xiaoling Zhang, Yuanyue Geng, Shengping Kang, Shixiao Xu

**Affiliations:** 1 Northwest Institute of Plateau Biology, Chinese Academy of Sciences, Xining 810001, Chin; 2 University of Chinese Academy of Sciences, Beijing 100049, China

**Keywords:** High-throughput Sequencing, Intestine, Microbiota, Qinghai Semi-fine Wool Sheep, Qinghai-Tibetan Plateau

## Abstract

**Objective:**

The intestinal microbiota enhances nutrient absorption in the host and thus promotes heath. Qinghai semi-fine wool sheep is an important livestock raised in the Qinghai-Tibetan Plateau; however, little is known about the bacterial microbiota of its intestinal tract. The aim of this study was to detect the microbial characterization in the intestinal tract of the Qinghai semi-fine wool sheep.

**Methods:**

The bacterial profiles of the six different intestinal segments (duodenum, jejunum, ileum, cecum, colon and rectum) of Qinghai semi-fine wool sheep were studied using 16S rRNA V3–V4 hypervariable amplicon sequencing.

**Results:**

A total of 2,623,323 effective sequences were obtained, and 441 OTUs shared all six intestinal segments. The bacterial diversity was significantly different among the different intestinal segments, and the large intestine exhibited higher bacterial diversity than the small intestine. Firmicutes, Bacteroidetes, and Patescibacteria were the dominant phyla in these bacterial communities. Additionally, at the genus level, *Prevotella_1*, *Candidatus_Saccharimonas*, and *Ruminococcaceae_UCG-005* were the most predominant genus in duodenal segment, jejunal and ileal segments, and cecal, colonic, and rectal segments, respectively. We predicted that the microbial functions and the relative abundance of the genes involved in carbohydrate metabolism were overrepresented in the intestinal segments of Qinghai semi-fine wool sheep.

**Conclusion:**

The bacterial communities and functions differed among different intestinal segments. Our study is the first to provide insights into the composition and biological functions of the intestinal microbiota of Qinghai semi-fine wool sheep. Our results also provide useful information for the nutritional regulation and production development in Qinghai semi-fine wool sheep.

## INTRODUCTION

The gastrointestinal microbiota of mammals is an extremely complex ecosystem, with a great diversity of bacteria, fungi, protozoa, and archaea [1]. For ruminants, the anaerobic microorganisms in rumen are used to convert feedstuff to short-chain, volatile fatty acids, which are absorbed by animal and used in energy metabolism and protein synthesis [2]. Comparatively, the intestines (including the small and large intestines ) are the most predominant location of the hundreds of millions of microbes, and the ruminal microbiota cannot reflect the core microbiota in other intestinal segments according to previous studies [3,4]. Several researches have demonstrated that intestinal micorbiota play a vital role in the biological degradation of the plant fibers, non-fiber carbohydrates, and polysaccharides [5,6]. Apart from these, the intestinal micorbiota also provide other benefical host functions, such as balancing the immune function, stabilizing the gut environment, and improving the health level in animals [7,8]. Microbiological studies of the different intestinal segments can be found in a few researches, such as in the camel, yak, and pig [6,9,10]. Most of these studies reported that the microbiota of the different intestinal segments had distinct characterization and specific biological functions.

The Qinghai semi-fine wool sheep is an ovine breed well-adapted to the harsh plateau environment in China. Additionally, Qinghai semi-fine wool sheep are the major economic resources for local pastoralists with many favorable advantages, such as fast growth traits, good wool traits, good meat flavor, and strong adaption [11]. It is therefore important to enhance relevant nutrition research to improve production performance and economic benefits of Qinghai semi-fine wool sheep. On the other hand, these excellent properties were not only correlated with maternal genetic effect but also with the intestinal microorganisms. However, information on the composition and biological functions of the intestinal microboiota of Qinghai semi-fine wool sheep remains unknown. With the ongoing development of the high-throughput sequencing technology and bioinformatics, dynamic changes and biological functions of the microbial community in the intestinal segments could be fully revealed.

In the present study, we used high-throughput sequencing based on the Illumina HiSeq2500 platform to analyze the microbial composition and functions of the six different intestinal segments (duodenum, jejunum, ileum, cecum, colon, and rectum) of the Qinghai semi-fine wool sheep. Through this research, we aim to explore microbial community structure characteristics of intestinal tract in Qinghai semi-fine wool sheep thoroughly and provide useful information for the nutritional regulation and production development.

## MATERIALS AND METHODS

### Sample collection and ethics approval

Three male, 1-year-old healthy Qinghai semi-fine wool sheep (body weight 35.7±1.23 kg) were obtained from a farm located in Wulan Country (Qinghai Province, China). The sheep were fed the same diet under the standard management practices. The diet contained 50.0% oat hay, 16.5% corn, 12.0% wheat, 7.5% wheat bran, 5.5% soybean meal, 6.0% rapeseed dregs, 0.50% NaCl, 0.3% CaHPO_4_, and 1.7% premix. Then, the sheep were slaughtered, and the fresh content samples (10 g) from the six intestinal segments, namely, the duodenum (Duo1, Duo2, and Duo3), jejunum (Jej1, Jej2, and Jej3), ileum (Ile1, Ile2, and Ile3), cecum (Cec1, Cec2, and Cec3), colon (Col1, Col2, and Col3), and rectum (Rec1, Rec2, and Rec3) were collected. The samples were immediately frozen in liquid nitrogen and stored at −80°C. The animal procedures in this study were performed according to the Experimental Animal Use Ethics Committee of the Northwest Institute of Plateau Biology, CAS (Permit Number: NWIPB20160302).

### DNA extraction and bacterial 16S rRNA sequencing

Total genomic DNA was extracted from each sample using a QIAamp Fast DNA Stool Mini Kit (QIAGEN, Hilden, Germany) following the manufacturer’s instructions. The quality of the DNA was visually assessed using 1.0% (w/v) agarose gel electrophoresis, and the DNA concentration was determined using a NanoDrop spectrophotometer (Nano Drop Technologies, Wilmington, DE, USA). The V3–V4 region of the 16S rRNA gene was amplified from genomic DNA using polymerase chain reaction (PCR) (95°C for 2 min, followed by 25 cycles at 95°C for 30 s, 55°C for 30 s, 72°C for 30 s, and a final extension at 72°C for 5 min) with the primers 338F (ACTCCTACGGGAGGCAGCA) and 806R (GGACTACHVGGGTWTCTAAT). The PCR reactions were performed in a triplicate 25 μL reaction system containing 2 μL DNA template, 2.5 μL 10× TranStart Taq buffer, 1 μL forward and reverse primer respectively, 2 μL dNTPs (2.5 mM), 0.25 μL TransStart Taq DNA polymerase, and 16.25 μL ddH_2_O. The PCR products were purified with a DNA Purification Kit (TIANGEN, Beijing, China), and barcoded V3–V4 amplicons were sequenced using the Illumina HiSeq platform (HiSeq2500 PE250).

### Bioinformatics analysis

The QIIME Pipeline (Version 1.8.0) was used to analyze the raw reads. Low-quality sequences were removed. High-quality sequences were binned into operational taxonomic units (OTUs) based on 97% sequence similarity, and the most abundant sequence within each OTU was identified as the representative sequence. Alpha diversity indices (ACE, Chao 1, Shannon, and Simpson indices) were calculated using QIIME. For beta diversity, the variations in the microbial composition among groups were investigated using the weighted UniFrac distance method, and this distance was presented using principal coordinate analysis (PCoA). The significance of the fractions in the PCoA plot was tested using analysis of similarities (ANOSIM). The LEfSe software (LEfSe 1.0) was used to analyze the differences in bacterial microbiota among groups, which defaulted to a filter value of 4 for the liner discriminant analysis (LDA) score. The biological functions of the bacterial microbiota were predicted using Phylogenetic Investigation of Communities by Reconstruction of unobserved states (PICRUSt) in combination with Kyoto encyclopedia of genes and genomes (KEGG) data.

### Statistical analysis

One-way analysis of variance and Tukey’s post hoc tests were conducted by using IBM SPSS 22.0 (SPSS Inc., Chicago, IL, USA). The data are presented as mean±standard deviation, and the significance was set at p<0.05.

## RESULTS

### Sequencing data

In this study, the hypervariable V3–V4 region of the 16S rRNA gene sequence analysis of 18 intestinal samples generated 2,623,323 effective sequences, with an average of 145,740 sequences per sample. A total of 1,210 OTUs were detected based on the 97% sequence similarity, and the number of OTUs for the duodenum, jejunum, ileum, cecum, colon, and rectum groups were 800, 765, 1,001, 1,046, 1,112, and 998, respectively. Furthermore, 441 OTUs were found in all six intestinal segments. The sequences were assigned to 15 phyla, 24 classes, 33 orders, 68 families, and 186 genera.

### Diversity of the intestinal microbiota

To compare the diversity and richness of the bacterial species among the different intestinal segments, the ACE, Chao1, Shannon, Simpson index, and Good’s coverage were calculated (Table 1). It was found that the Good’s coverage of each sample was more than 99.8%, indicating that the sequencing depth was sufficient to access the microbial diversity. The cecum and colon samples owned the highest ACE and Chao 1 richness values (p<0.05). In contrast, the jejunum samples possessed the lowest richness values (p<0.05). For community diversity comparison, the ileum samples had the highest Simpson values (p<0.05), and the rectum samples had the lowest Simpson values (p<0.05).

**Figure 1:**
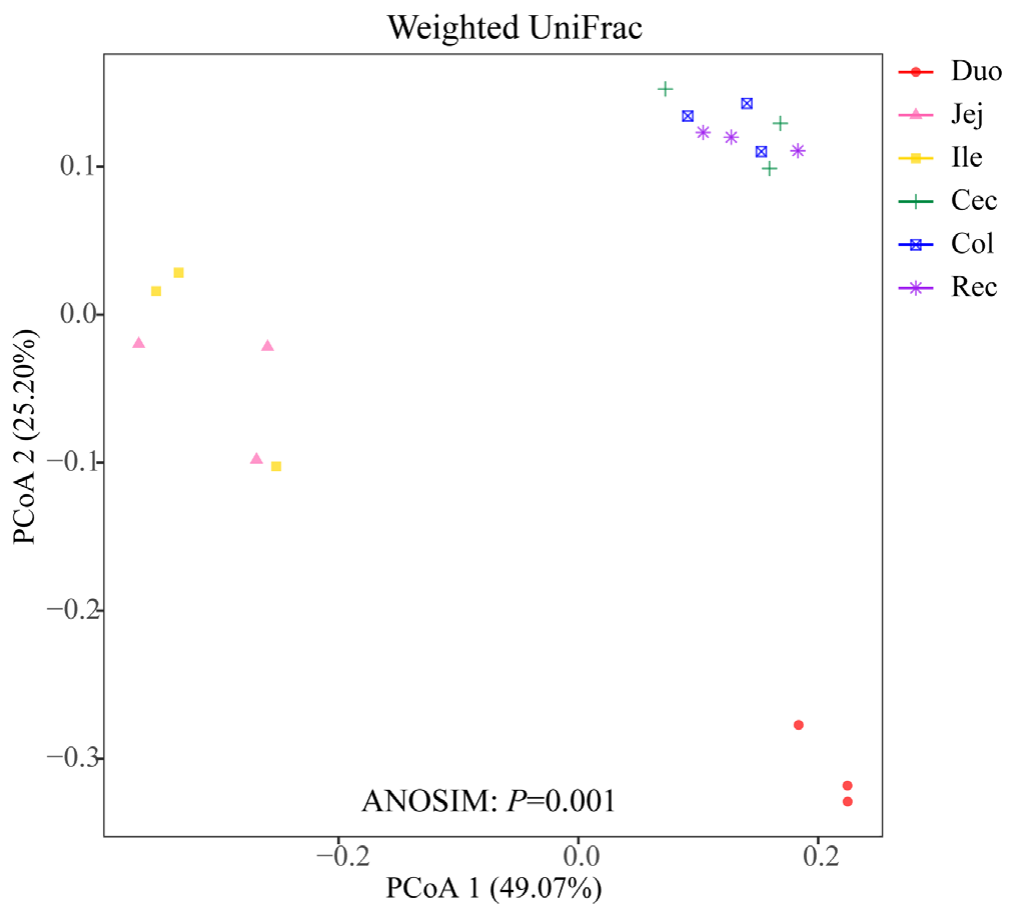
Principal coordinate analysis (PCoA) of microbial communities based on weighted UniFrac distance.

To compare the microbial community among the different intestinal segments, PCoA plot based on the weighted UniFrac distance matrices was employed (Figure 1). The ANOSIM result based on the distance matrice showed significant differences in the microbial communities of the six intestinal segments (p = 0.001). The three samples in the duodenum group were clearly distinguished from those of the other intestinal segments. Moreover, the samples in the cecum, colon, and rectum groups were concentrated in spatial location.

### Composition and structure of the intestinal microbiota

**Figure 2:**
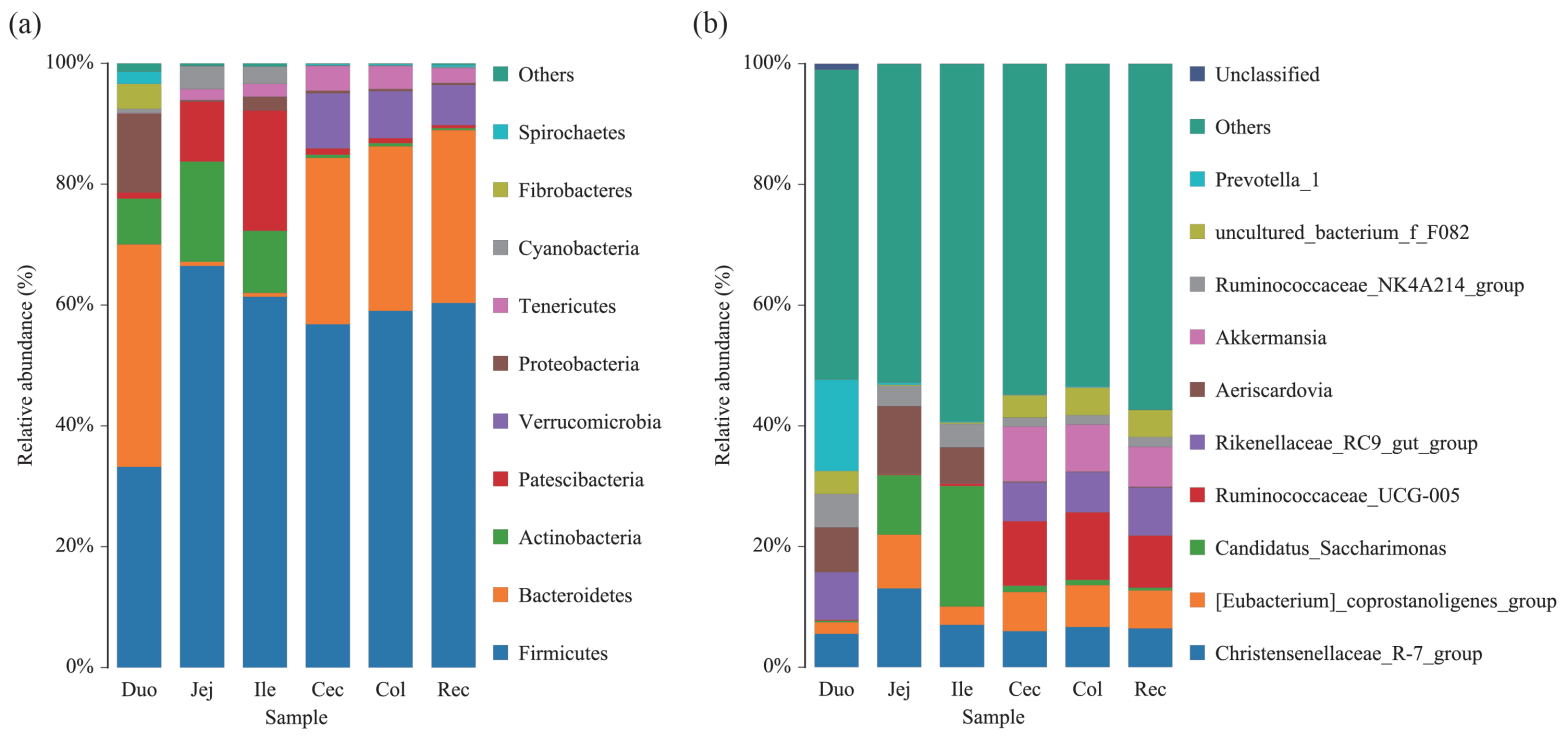
Composition and structure of the bacterial microbiota across the different intestinal segments at the (a) phylum level and (b) genus level.

At the phylum level, 15 phyla were detected via taxonomic analysis. Figure 2a shows the top 10 most abundant phyla in all groups. In the duodenum group, Firmicutes, Bacteroidetes, Proteobacteria, Actinobacteria, and Fibrobacteres were the most abundant phyla, representing 33.40%, 39.00%, 9.52%, 7.98%, and 3.87% of the total reads, respectively. Within the jejunum and ileum groups, Firmicutes (abundances 63.70% and 61.00%, respectively), Actinobacteria (15.70% and 9.82%, respectively), Patescibacteria (13.10% and 20.60%, respectively), and Cyanobacteria (4.00% and 2.81%, respectively) were the dominant phyla. The dominant phyla in the cecum, colon, and rectum groups were similar; Firmicutes (abundances 57.90%, 59.30%, and 60.20%, respectively) was the most abundant phylum, followed by Bacteroidetes (28.20%, 27.30% and 28.70%, respectively), Verrucomicrobia (7.94%, 7.49%, and 6.75%, respectively), Tenericutes (3.41%, 3.59%, and 2.54%, respectively), and Patescibacteria (1.18%, 0.90%, and 0.48%, respectively).

At the genus level, 186 genera were detected in the six intestinal segments of Qinghai semi-fine wool sheep. Figure 2b shows the top 10 abundant genera in all groups. In the duodenum group, *Prevotella_1*, *Rikenellaceae_RC9_gut_group*, and *Succinivibrionaceae_UCG-002* were the most abundant genera, representing 14.60%, 8.53%, and 8.20% of the total reads, respectively. However, the most abundant genus in the jejunum and ileum groups was *Candidatus_Saccharimonas* (13.10% and 20.60%, respectively). Within the cecum, colon, and rectum groups, *Ruminococcaceae_UCG-005* (11.10%, 11.30%, and 8.63%, respectively) was the dominant genus.

### Linear discriminant analysis of the intestinal microbiota

**Figure 3:**
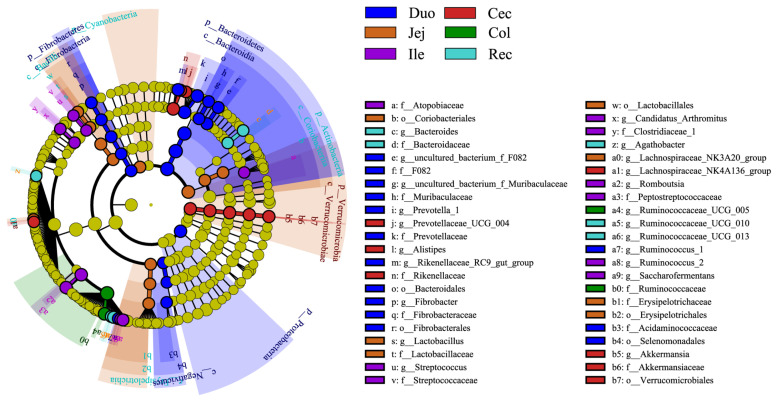
Linear discriminant analysis effect size (LEfSe) cladogram showing the taxonomic differences among the different intestinal segments (liner discriminant analysis >4, p<0.05).

We performed LDA coupled with effect size measurements (LEfSe) analysis to identify which were significantly different between the different intestinal segments (LDA>4, p<0.05). The results showed that 57 different bacterial taxa were found among the six intestinal segments (Figure 3). LEfSe showed that 20 bacterial taxa were significantly abundant in the duodenum group (e.g., Bacteroidales and Bacteroidetes), 12 bacterial taxa were significantly abundant in the jejunum group (e.g., Actinobacteria and Lactobacillales), 9 bacterial taxa were significantly abundant in the ileum group (e.g., Peptostreptococcaceae and *Romboutsia*), 9 bacterial taxa were significantly abundant in the cecum group (e.g., Rikenellaceae and Verrucomicrobiales), 2 bacterial taxa were significantly abundant in the colon group (e.g., Ruminococcaceae and *Ruminococcaceae_UCG_005*), and 5 bacterial taxa were significantly abundant in the rectum group (e.g., *Ruminococcaceae_UCG_013* and *Agathobacter*).

### Predicted biological functions of intestinal microbiota

**Figure 4:**
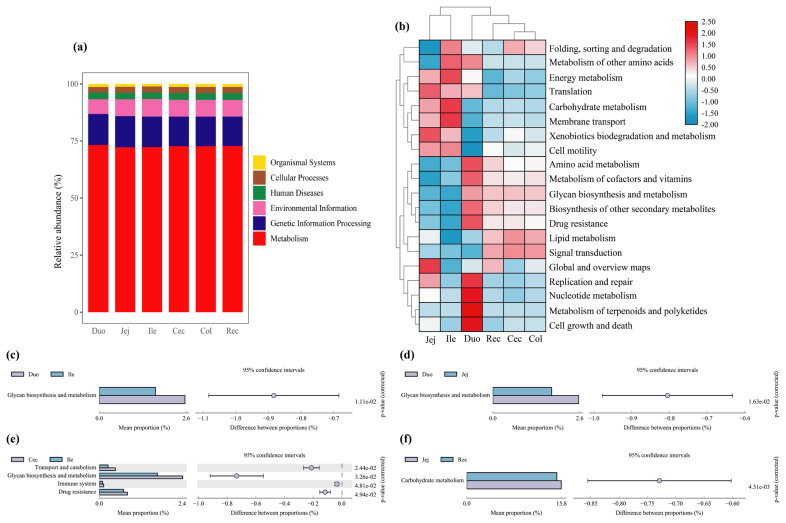
Microbial functional predictions. (a) Distribution of the dominant functions genes based on KEGG level 1; (b) Heatmap of the top 20 functional gene distributions based on KEGG level 2; (c)–(f) Comparisons of the five significantly different functional genes of the microbiota across the six intestinal segments. KEGG, Kyoto encyclopedia of genes and genomes.

To investigate the biological functions of the bacterial microbiota across the different intestinal segments, we predicted the microbial functions using PICRUSt. At KEGG level 1, it showed that 6 gene families were present in the different intestinal samples, and the category “Metabolism” had the highest relative abundance, with more than 72% of total reads in each group (Figure 4a). At KEGG level 2, 43 gene families were identified in all samples; the majority of the genes belonged to carbohydrate metabolism, global and overview maps, amino acid metabolism, energy metabolism, and metabolism of cofactors and vitamins. For clarity and visualization purposes, the relative abundances of the top 20 gene families are shown in a heatmap (Figure 4b), which revealed that the large intestinal groups (including the cecum, colon, and rectum groups) clustered together and had similar biological functions, whereas the jejunum and ileum groups clustered together. To better understand the differences among the gene families across the different intestinal segments, we compared the relative abundance of these 43 gene families. The results showed that only 5 gene families were significantly different across the different intestinal segments (p< 0.05) (Figure 4c–f). The abundance of genes involved in glycan biosynthesis and metabolism was higher in the duodenum group than in the jejunum and ileum groups (p<0.05). In addition, the proportions of gene families involved in transport and catabolism, glycan biosynthesis and metabolism, immune system and drug resistance were higher in the cecum group than in the ileum group (p<0.05). For carbohydrate metabolism, the abundance of genes was higher in the jejunum group than in the rectum group (p<0.05).

## DISCUSSION

The intestinal microbiota is commonly known as the second genome of the animal body and can provide several benefits to the host. Thus, an investigation of the microbial community structure characteristics and biological functions in all intestinal segments are necessary. The objective of this study was to analyze the composition and biological functions of the bacterial microbiota in the six different intestinal segments (including duodenum, jejunum, ileum, cecum, colon, and rectum) of the Qinghai semi-fine wool sheep using next-generation sequencing technology.

Many studies have previously been carried out on the bacterial microbiota of the intestinal segments of ruminants. Zhang et al [5] analyzed the bacterial composition of the intestinal segments of small-tail Han sheep, and the results showed that the cecum and rectum owned a higher diversity of microbiota than the jejunum. Zeng et al [12] studied the microbial composition of the intestinal segments of Chinese Mongolian sheep and reported that the bacterial diversity was higher in the large intestine than in the small intestine. In the present study, we analyzed the diversity and richness of the bacterial species in the intestinal samples of the Qinghai semi-fine wool sheep by calculating the ACE, Chao1, Shannon, and Simpson indices, and the results showed that the cecum and colon had the highest ACE and Chao 1 richness values, which is consistent with previous studies on the low-elevation sheep breed. In addition, a recent study reported that the duodenum, jejunum, and ileum of yak possess a relatively similar microbiota composition, and these samples clustered together based on the PCoA analysis [6]. Using the PCoA analysis based on the weighted UniFrac distance matrice, we obtained similar results in this study. The samples from the cecum, colon, and rectum were clustered together, and three samples in the duodenum group were clearly differentiated from the samples of the other intestinal segments.

Our data revealed that the dominant phyla in the intestinal segments of the Qinghai semi-fine wool sheep were composed of Firmicutes and Bacteroidetes, which play an important role in the digestion of carbohydrates and proteins in mammals [13,14]. Similarly, these two dominant phyla were found in the intestinal tracts of yak [15], sheep [5,16], and steer [17]. This indicates the important ecological functions of these two phyla in the ruminal intestinal segments. Previous studies have demonstrated that Firmicutes can promote the digestion of fiber and cellulose [18,19]. In this study, Firmicutes was the dominant phylum (constituting 57.90% to 63.70% of the total bacterial reads) in the jejunum, ileum, cecum, colon, and rectum. However, the composition of the bacterial microbiota in the duodenum was different from that of other intestinal segments, and this result was consistent with the result of the PCoA analysis. Interestingly, the relative abundances of Bacteriodetes and Firmicutes in the duodenum were maintained at the same level in the present study. We speculated that this special characteristic of bacterial composition was caused by the special location of the duodenum, which connects the stomach and latter intestinal segments. Due to the proximal region to the stomach, the duodenum had a lower pH value than other intestinal segments and lower bacterial counts [20]. For the physiological function, several studies revealed that the duodenum was important for food digestion and nutrient absorption, especially for the absorption of glucose [21]. Thus, special location and biological function are responsible for the special microbial communities in duodenum. Furthermore, Bacteriodetes can promote the digestion of proteins and polysaccharides [14]. In this study, we found that the relative abundance of Bacteriodetes tended to decrease in the jejunum (0.68%) and ileum (0.67%). This is consistent with studies on the microbial community across the intestinal segments of dairy cattle [3]. However, the relative abundance of Actinobacteria showed a tendency to increase in these two segments. Previous results showed that Actinobacteria was the predominant phyla in the jejunum of Mongolian horses [22]. Besides, Actinobacteria is the prevalent phyla in the gut of human and considered to be linked with atherogenic lipid metabolites and proinflammatory cytokines [23–25]. A recent study showed that the prevalence of Verrucomicrobia was observed not only in the ileum but also in the large intestine of camels [10]. Researchers have also shown that Verrucomicrobia can be conducive for polysaccharide degradation and methane oxidation [26]. In our study, a higher abundance of Verrucomicrobia was only observed in the large intestine of the Qinghai semi-fine wool sheep. This indicates that the large intestine could improve food utilization by strong secondary digestion of food for ruminants.

In addition, this study showed that the genus *Prevotella_1* was the most abundant bacteria in the duodenum of the Qinghai semi-fine wool sheep. This result is in agreement with previous studies [27,28]. *Prevotella_1* plays an important role in dissolving proteins and carbohydrates, such as starch and pectin, and is enriched in the rumen of ruminants [29]. Our results also indicated that the relative abundance of the genera *Candidatus_Saccharimonas* and *Christensenellaceae_R-7_group* was higher in the jejunum and ileum than in the other intestinal segments. *Candidatus_Saccharimonas* is affiliated with the phylum Saccharibacteria, and is one of the dominant genera in the jejunum of goat [30]. Previous study showed that the *Candidatus* Saccharibacteria was closely associated with the cellulose degradation and utilization in the rumen of beef cows [31]. This may also implicates the small intestine as an important site for the digestion of feed. *Christensenellaceae_R-7_group* is affiliated with the family Christensenellaceae and phylum Firmicutes and seems to play an important role in health [32]. Relevant study showed that the Christensenellaceae was related to the body weight in humans and mice, and could regulate lipid metabolism and reduce prevalence of obesity [33]. However, the top three genera in the large intestines (cecum, colon, and rectum) were *Ruminococcaceae_UCG-005*, *Akkermansia*, and *Rikenellaceae_RC9_gut_group*. It is known that *Ruminococcaceae* is widely distributed in the rumen and large intestine of ruminants, which play an important role in dissolving cellulose, hemicellulose, and starch [34,35]. In addition, the genus *Akkermansia* is affiliated with the phylum Verrucomicrobia and inhabits the intestinal tract of the Bactrian camel [10]. Previous studies have reported that *Akkermansia* is involved in the reduction of obesity, inflammation, and diabetes in mice. Additionally, *Rikenellaceae_RC9_gut_group* is an abundant bacteria in the rumen, reticulum, and abomasum of cattle and yak [15,36]. It has been reported that the *Rikenellaceae_RC9_gut_group* is closely connected to the utilization of carbohydrates and nitrogen in the large intestine of ruminants [15].

Based on the predicted PICRUSt of intestinal bacteria, at the KEGG level 1, metabolism, genetic information processing, environmental information, human diseases, cellular processes, and organismal systems were the typical microbial functions in the Qinghai semi-fine wool sheep. Among them, metabolism was the most abundant in our study, in agreement with the previous studies in cattle [3] and pigs [9]. Furthermore, many pathways related to metabolism (e.g., carbohydrate, energy, amino acid, lipid, and nucleotide metabolism) were detected at the KEGG level 2, and significant differences in the bacterial functions among the intestinal segments of the Qinghai semi-fine wool sheep were observed. For example, the duodenum carried out more glycan biosynthesis and metabolism than the ileum and jejunum, which was related to the higher relative abundance of Bacteroidetes in the duodenum. In the present study, we detected that transport and catabolism, immune system, and drug resistance pathways were significantly more abundant in the cecum than in the ileum. In addition, the pathway related to carbohydrate metabolism was more abundant in the jejunum than that in the rectum. These finding were consistent with those of previous studies investigating the general functions of the intestinal microbiota of Tan sheep [16]. These results also indicate that the small intestine is mainly responsible for the digestion and absorption of nutrients as an important digestive organ in the Qinghai semi-fine wool sheep.

## CONCLUSION

This study mainly revealed the intestinal bacterial composition and potential biological functions in the Qinghai semi-fine wool sheep based on 16S rRNA V3–V4 hypervariable amplicon sequencing. Distinct differences in both microbial diversity and relative abundance were observed among the different intestinal segments. The present study also indicated that the different intestinal segments were characterized by special biological functions. Besides, the microbial characteristics of intestinal tract in Qinghai semi-fine wool sheep, including the microbial diversity, dominant bacteria species, and potential biological functions, were in accordance with the low-elevation sheep breed (e.g. small-tail Han sheep, Chinese Mongolian sheep, and Tan sheep). These results may improve our understanding of the role of intestinal microbiota role in Qinghai semi-fine wool sheep. Furthermore, the data would also provide useful information for the nutritional regulation and production development in Qinghai semi-fine wool sheep.

## Figures and Tables

**Table 1:** Statistical analysis of alpha diversity

Region	ACE	Chao1	Shannon	Simpson	Good’s coverage
Duo	727.78±110.73^ab^	678.10±39.08^bc^	4.45±0.47	0.038±0.030^ab^	0.998±0.001^b^
Jej	652.50±185.04^b^	636.26±137.38^c^	4.08±0.11	0.061±0.015^a^	0.999±0.000^a^
Ile	849.23±14.73^ab^	849.31±26.04^abc^	3.99±0.35	0.061±0.025^a^	0.999±0.000^ab^
Cec	946.70±29.27^a^	965.08±34.95^a^	5.12±0.54	0.021±0.015^b^	0.999±0.000^ab^
Col	944.85±126.48^a^	953.77±123.26^a^	5.12±0.63	0.022±0.017^b^	0.999±0.000^a^
Rec	888.21±53.28^ab^	891.92±67.46^ab^	5.21±0.46	0.017±0.011^b^	0.999±0.000^a^
p-value	0.0243	0.0013	0.0151	0.0496	0.0289

Alpha diversity index is expressed as the mean±standard deviation.

Duo, duodenum; Jej, jejunum; Ile, ileum; Cec, cecum; Col, colon; Rec, rectum.

a–c Significant differences are indicated by different letters (p<0.05) in the same column.
